# Long-term effects of different planting patterns on greenhouse soil micromorphological features in the North China Plain

**DOI:** 10.1038/s41598-019-38499-6

**Published:** 2019-02-18

**Authors:** Zhe Liu, Jichang Han, Zenghui Sun, Tianqing Chen, Yin Hou, Na Lei, Qiguang Dong, Jing He, Yizhong Lu

**Affiliations:** 1 Shaanxi Province Land Engineering Construction Group, Key Laboratory of Degraded and Unused Land Consolidation Engineering, the Ministry of Land and Resources of China, Xi′an, China; 2 College of Resources and Environmental Science, China Agricultural University, Beijing, China; 3 Shaanxi Provincial Land Consolidation Engineering Technology Research Center, Xi′an, China

## Abstract

Soil structure represents a basis for soil water retention and fertiliser availability. Here, we performed a micromorphological analysis of thin soil sections to evaluate the effects of 10 years of organic planting (OPP), pollution-free planting (PFP), and conventional planting (CPP) on greenhouse soil structure in the North China Plain. We also analysed soil bulk density, soil organic matter (SOM), and wet aggregate stability. The CPP soil microstructure included weakly separated angular block or plate forms and weak development of soil pores (fissured or simply accumulated pores) with the highest bulk density (1.33 g cm^−3^) and lowest SOM (26.76 g kg^−1^). Unlike CPP, the OPP soil microstructure was characterised by highly separated granular and aggregated structures and an abundance of plant and animal remains. OPP was associated with the highest total porosity (55.4%), lowest bulk density (1.17 g cm^−3^), and highest SOM (54.81 g kg^−1^) in the soil surface layer. OPP also improved the ventilation pore content (proportion of pores >0.1 mm, 44.09%). OPP aggregates showed different hierarchies of crumb microstructure and higher mean weight diameter and geometric mean diameter values than did CPP. These results confirm the benefits of long-term OPP for soil structure and quality in the greenhouse.

## Introduction

Soil is a porous medium formed by the connection of solid components and pores in different sizes and shapes, representing the most complex biological material on the earth. Research on soil structure has mainly focused on soil solids and soil pores^1,2^. The processes of soil shrinkage, dispersion, and agglomeration are important structural process, which occur due to soil water, gas, thermal movement, and material transformation (such as carbon and nitrogen). Soil structural degradation is the main contributor to general soil degradation. The most obvious feature of degradation is a reduction in the soil organic matter content and the stability of soil aggregates^3–6^. The study of micromorphology is an important issue in the field of soil science and is an essential part of studying soil characteristics.

Soil micromorphology is a new research area in soil science that has attracted significant attention from scholars; this approach uses image processing technology to quantitatively analyse the quantity and size of pores and soil forming materials in soil sheets^7–9^. The evolution of soil micromorphology characteristics can reflect the relationship between soil formation and development and the environment, which can better inform the role of human factors in changing soil structure^10–12^.

Soil structure is the basis for water and nutrient retention properties. A large number of studies have shown that sustainable planting methods can increase soil organic matter content and improve soil structure, thereby improving soil quality and productivity. In one study, organic planting patterns were conducive to the formation of soil aggregates and the stabilisation of soil structure^13–15^. To date, the study of soil micromorphology has mostly focused on the micromorphology of soil formation classification, the micromorphology of regional soil, and the study of paleosol micromorphology^11,16,17^. Zhou *et al*.^18^ used petrographic microscopy (Olympus BX51-P) to observe and quantitatively analyse thin slices of black soil under different tillage practices in Northeast China and identified positive effects of long-term no tillage practices on formation of a stable soil structure. Pang *et al*.^19^ studied the characteristics of Loess-paleosol micromorphology and confirmed that pedogenesis was impeded during the mid-Holocene in the Middle Yellow River. Zanuzzi *et al*.^20^ analysed the micromorphological characteristics of unamended and amended organic and industrial wastes and concluded that organic amendments were better for the formation of granular microstructure in mined areas. Khademi *et al*.^11^ studied the micromorphological characteristics of the three different horizons of Iranian gypsiferous aridisols and validated the utility of the American Soil Classification System in this context.

The North China Plain is an important crop production area in China, but the long-term heavy use of chemical fertilisers and unreasonable cultivation methods have caused problems with soil quality and structural degradation, which negatively affect crop production^21,22^. Accordingly, the sustainable cultivation of soils in the North China Plain is very important, yet few studies have compared the characteristics of greenhouse soil microstructure and physicochemical properties under different planting patterns from a micromorphological perspective. The long-term study of different planting patterns is important for studying soil quality and structural evolution, and can be used to systematically identify issues and trends in soil quality and structure as well as nutrient cycling and balance^23^. Studying soil micromorphology under different planting patterns can delineate specific effects on the morphology of soil pores, soil forming materials, and the composition, spatial distribution, morphology, and structure of soil particles, and inform the relationship between parent material minerals and organisms in soil^24,25^. Therefore, we compared the long-term effects of different planting patterns on soil micromorphology, soil physicochemical properties, the evolution of soil quality, and typical greenhouse soil structure in the North China Plain in order to provide a theoretical basis for improving soil structure and soil quality in the region.

## Results

### Effect of different planting patterns on soil bulk density, soil organic matter, and water-stable aggregates

The bulk density (BD) under OPP was significantly lower than that under PFP and PFP (Table 1, *P* < 0.05). The order of BD was OPP < PFP < CPP for the three soil depth layers. In the 0–10 cm soil layer, the BD under OPP was 12.0% and 8.6% less than that under CPP and PFP, respectively; in the 10–20 cm and 20–40 cm soil layers the BD showed the same decreasing trend, the BD under OPP was 9.8% and 3.0% (10–20 cm soil layer) and 5.8% and 4.6% (20–40 soil layer) less than that under CPP and PFP, respectively. These results indicated that long-term OPP reduced soil compaction and yielded a lower BD in the surface layer than in the bottom layer.

**Table 1:** Soil bulk density, organic matter, and *R*_0.25_ at different depths under different planting patterns.

Depth	BD (g cm^−3^)			SOM (g kg^−1^)			*R*_0.25_ (%) (wet sieving)		
(cm)	CPP	PFP	OPP	CPP	PFP	OPP	CPP	PFP	OPP
0–10	1.33 ± 0.04aC	1.28 ± 0.04aC	1.17 ± 0.03bC	26.76 ± 2.11cA	40.40 ± 3.53bA	54.81 ± 5.43aA	15.2cA	27.1bA	30.8aA
10–20	1.43 ± 0.04aB	1.33 ± 0.04bB	1.29 ± 0.07bB	18.30 ± 1.42bB	19.90 ± 4.09bB	33.52 ± 4.64aB	9.5bB	16.4aB	17.8aB
20–40	1.56 ± 0.08aA	1.49 ± 0.06abA	1.45 ± 0.01bA	6.75 ± 0.59bC	7.24 ± 0.78bC	11.09 ± 2.50aC	3.5bC	3.9bC	7.0aC

BD, soil bulk density; SOM, soil organic matter; *R*_0.25_, >0.25 mm aggregates. CPP, conventional planting pattern; PFP, pollution-free planting pattern; OPP, organic planting pattern. Different lowercase letters represent significant differences between different planting conditions in the same soil layer. Different uppercase letters represent significant differences between different soil depths.

Soil organic matter (SOM) content under OPP was significantly higher than that under CPP and PFP. The order of SOM content was OPP > PFP > CPP for the three soil depth layers (Table 1, *P* < 0.05). In the 0–10 cm soil layer, SOM content under OPP was 104.8% and 35.7% more than that under CPP and PFP, respectively; the SOM content under OPP was 94.1% and 68.4% (10–20 soil layer) and 64.3% and 53.2% (20–40 soil layer) more than that under CPP and PFP, respectively.

Soil water-stable aggregates from wet sieving contribute more to the stability of soil structure and are much more important than unstable aggregates; therefore, we selected macroaggregates to investigate changes in soil aggregation, structure, and stability. Numbers of macroaggregates under OPP were significantly higher than those under CPP and PFP at depths of 0–10 and 20–40 cm, but not at 10–20 cm (Table 1). In the 0–10 cm soil layer, the *R*_0.25_ under OPP was 102.6% and 13.7% higher than that under CPP and PFP. These results indicated that long-term OPP not only increased SOM, but also increased macroaggregates.

### Effect of planting patterns on soil microstructure

**Figure 1:**
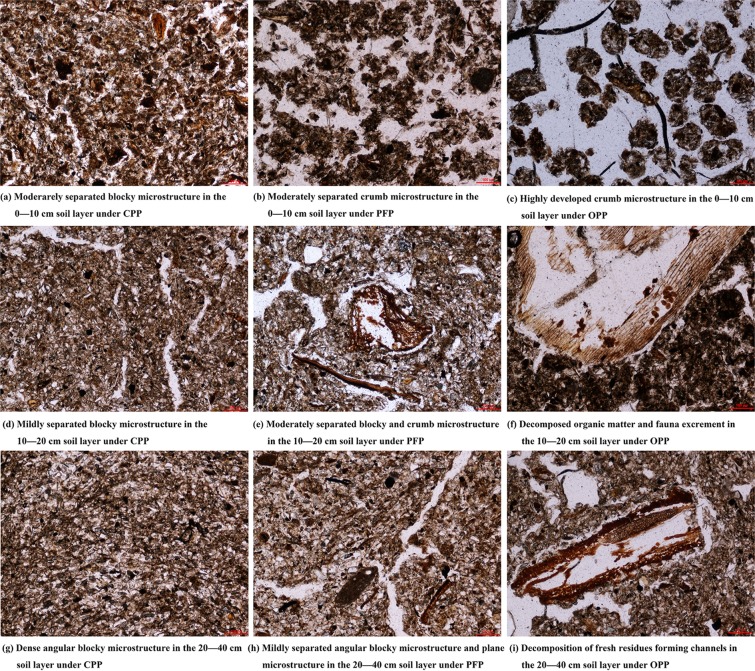
Soil microstructure under different planting conditions. Images are 1.10 mm × 0.76 mm, images were 50× magnification. CPP, conventional planting pattern; PFP, pollution-free planting pattern; OPP, organic planting pattern.

Based on soil sections and thin sections, soils from the three different planting conditions were Ap1-Ap2-AB; however, the characteristics of soil microstructure varied significantly (Table 2). Under CPP, the soil microstructure included moderately separated blocks and weakly separated plates in the 0–20 cm soil layer. The main pore types were surface cracks and simple accumulation pores (Fig. 1a,d). The pore wall was rough (*R* = 1.94) and individual pores were relatively small (*D* = 12.4 μm). Very few fresh plant residues, organic matter, and soil biological activities were found in the soil. Soil in the 20–40 cm layer was compact and the main type of soil microstructure was a dense and weakly separated block structure (Fig. 1g). The pore wall was rough (*R* = 1.69) and individual pores were small (*D* = 8.7 μm). Micromorphological observations and image analysis in this study showed that very few fresh plant residues and soil biological activities were found in the soil (Fig. 1a,d,g). These findings indicated that CPP was not conducive to the formation of soil aggregates or improvement of soil structure.

**Table 2:** Soil morphological characteristics under different planting patterns.

Planting pattern	Depth (cm)	Horizon	Colour and texture	Microstructure	Organic matter and soil organisms	Main pore type	Grain (μm, g kg^−1^)		
							2000~50	50~2	<2
CPP	0–10	AP1	Dark reddish brown, 10YR4/4, silty loam	Moderately separated angular blocky microstructure	Small amounts of fresh plant residue and soil fauna excrement	Fractures and simple packing voids *R* = 1.94, *D* = 12.4	129	629	242
	10–20	AP2	Yellowish brown, 10YR5/4, silty loam	Mildly separated blocky microstructure	Small amounts of plant residue cross-sections and plant residue fragments, very little organism activity	Plane microstructure *R* = 1.86, *D* = 11.6	139	638	223
	20–40	AB	Yellowish brown, 10YR5/4, silty loam	Dense angular blocky microstructure	Very few plant residues and little organism activity	Fractures *R* = 1.68, *D* = 10.8	95	703	202
PFP	0–10	AP1	Dark brown, 7.5YR3/4, silty loam	Moderately separated blocky and crumb microstructure	Moderate amounts of plant residues and soil fauna excrement	Vugh and dendritic pores, few compound packing voids *R* = 2.16, *D* = 16.8	136	715	149
	10–20	AP2	Brown, 7.5YR4/3, silty loam	Moderately separated blocky microstructure and mildly separated crumb microstructure	Small amounts of fresh plant residue and organism activity	Plane microstructure and simple packing voids *R* = 1.99, *D* = 14.2	158	623	219
	20–40	AB	Brown, 7.5YR4/4, silty loam	Mildly separated angular blocky microstructure and little crumb microstructure	Small amounts of animal and plant residues in semi-decomposition	Plane microstructure *R* = 1.92, *D* = 11.4	170	613	217
OPP	0–10	AP1	Dark reddish brown, 5YR3/4, silty loam	Highly developed crumb microstructure	Abundant fresh plant residue. Medium amounts of organism activities and soil fauna excrements	Compound packing voids, medium amounts of vugh and channels *R* = 2.57, *D* = 19.4	143	695	162
	10–20	AP2	Dark brown, 7.5YR3/4, silty loam	Moderately separated crumb and blocky microstructure	Medium amounts of plant residue, soil fauna excrements, and organism activities	Compound packing voids *R* = 2.29, *D* = 17.8	134	650	217
	20–40	AB	Brown, 7.5YR4/3, silty loam	Moderately separated blocky microstructure and mildly separated angular blocky microstructure	Little amounts of fresh plant residue and soil fauna excrements	Plane microstructure and simple packing voids *R* = 2.08, *D* = 13.4	175	620	205

A, leaching layer; AB, transitional soil layer; B, deposition layer; CPP, conventional planting pattern; *D*, equivalent diameter (μm); PFP, pollution-free planting pattern; OPP, organic planting pattern; p, soil layer disturbed by farming or other measures; *R*, smoothness of pore wall = (perimeter × perimeter)/(4 × area × 1.064).

Under PFP, soil microstructure in the 0–20 cm layer included moderately separated masses and a few highly separated granular structures. The pore walls were relatively smooth (*R* = 2.16) and individual pores were relatively large (*D* = 16.8 μm). Soil in the 10–40 cm layer included moderately isolated block microstructures, moderately separated plate microstructures (Fig. 1e), weakly separated block structures, and a small number of plate-like microstructures. The pore wall was rough (*R* = 1.92) and individual pores were small (*D* = 11.4 μm). A small amount of fresh plant debris and traces of soil biological activity were found in the soil (Fig. 1e,h). Accordingly, PFP was associated with better soil biological activity, increased organic matter content, and improvements in soil structure.

Under OPP, the 0–20 cm layer was mainly composed of highly separated granular structures and moderately separated granular structures. The pore wall was smooth (*R* = 2.57) and individual pores were quite large (*D* = 19.4 μm). There was an abundance of fresh plant residues and some soil organism excretions were visible near fresh plant debris; additionally, there were traces of soil biological activity (Fig. 1c,f). The main types of soil microstructure in the 20–40 cm layer were moderately separated granule structures and moderately separated angular microstructures. The pore wall was smooth (*R* = 2.29) and individual pores were relatively large (*D* = 17.8 μm). There were some traces of soil biological activity and incompletely decomposed plant residues (Fig. 1i). The results of the image analysis of the soil thin sections showed many well-developed aggregates, fresh organic matter, and semi-decomposed plant residues in the soil slices, and there was a significantly higher number of pores formed by biodegradation and the decomposition of organic matter compared with other planting patterns (Fig. 1). These results showed that OPP was associated with increased organism and enzyme activity in the soil and contributed to the formation of well-developed aggregates, as well as improvements in soil structure.

### Effect of planting patterns on soil aggregate stability

**Figure 2:**
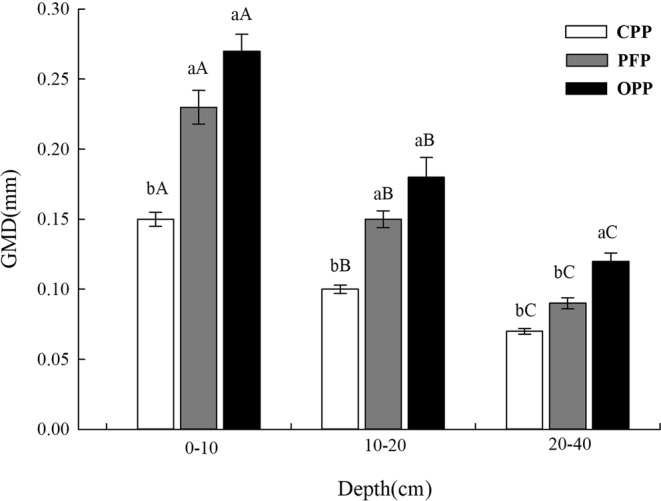
Effects of planting conditions on geometric mean diameter (GMD). A one-way analysis of variance and Duncan’s tests were used to compare planting conditions and different sample depths (α = 0.05). Error bars indicate the standard deviation. CPP, conventional planting pattern; PFP, pollution-free planting pattern; OPP, organic planting pattern.

**Figure 3:**
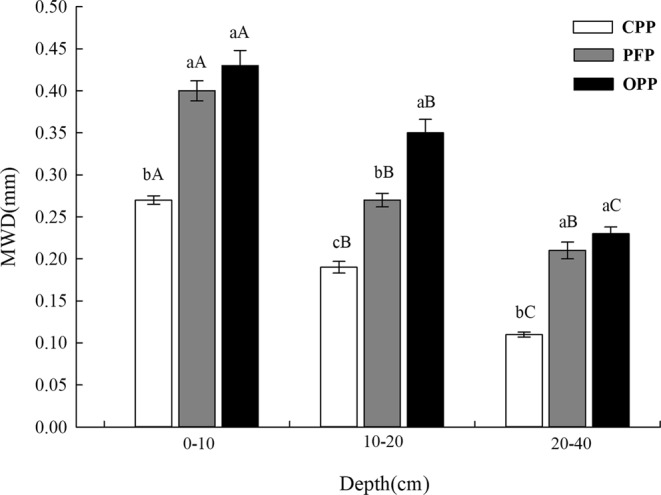
Effects of planting patterns on mean weight diameter (MWD). A one-way analysis of variance and Duncan’s tests were used to compare planting conditions and different sample depths (α = 0.05). Error bars indicate the standard deviation. CPP, conventional planting pattern; PFP, pollution-free planting pattern; OPP, organic planting pattern.

Soil aggregate stability measured by the wet sieving method revealed that the geometric mean diameter (GMD) and mean weight diameter (MWD) values of soil aggregates in the 0–10 cm, 10–20 cm, and 20–40 cm soil layers were best in the OPP, followed by the PFP and CPP (Figs 2 and 3; *P* < 0.05). Additionally, GMD and MWD values for the same planting pattern were higher in upper layers than in lower layers. In the 0–10 cm soil layer, GMD and MWD were 80.0% and 59.3% higher, respectively, under OPP compared with CPP. Under OPP, GMD and MWD showed the same increasing trend and those values were 80.0% and 84.2% (10–20 cm soil layer) and 71.4% and 109.1% (20–40 cm soil layer) higher, respectively, than under CPP. Similarly, GMD and MWD were 17.4% and 7.5% higher, respectively, under OPP than PFP in the 0–10 cm soil layer. Under OPP, GMD and MWD values were 20.0% and 29.6% (10–20 cm soil layer) and 33.3% and 9.5% (20–40 cm soil layer) higher, respectively, than under PFP.

### Effect of planting patterns on soil porosity

**Figure 4:**
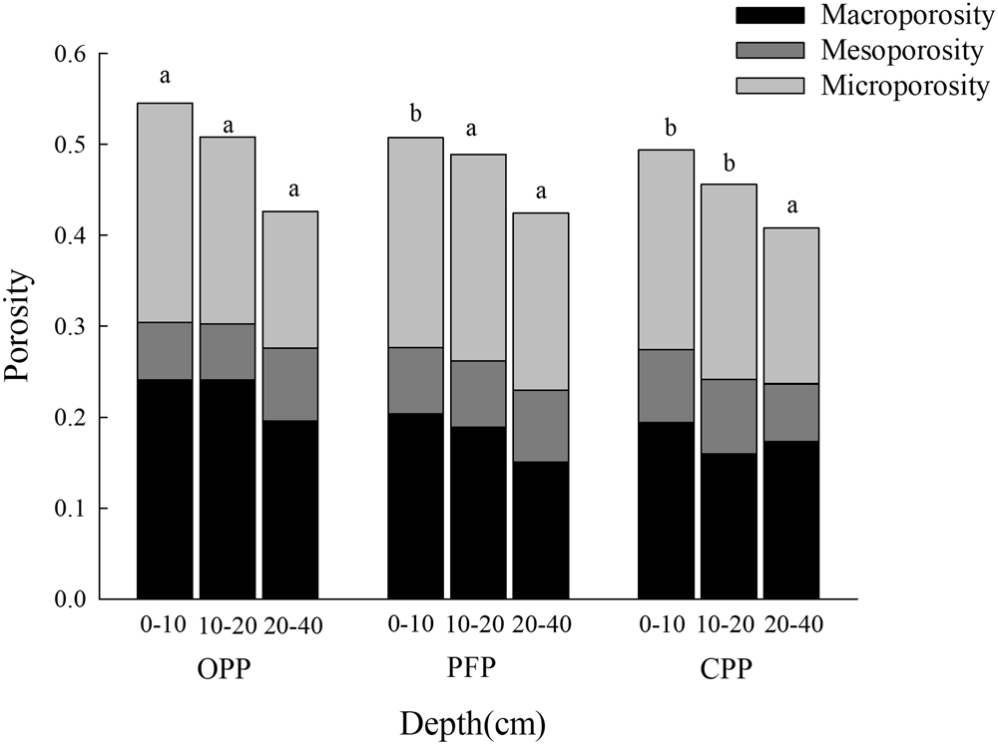
Effect of planting patterns on total porosity, microporosity (<20 µm), mesoporosity (20–100 µm), and macroporosity (>100 µm). A 1-way analysis of variance and Duncan’s tests were used to compare planting conditions at the same depths (α = 0.05). Total porosity values followed by the same letter were not significantly different at the 0.05 level. CPP, conventional planting pattern; PFP, pollution-free planting pattern; OPP, organic planting pattern.

In the 0–10 cm soil layer, the total porosity under OPP was significantly higher than that under PFP or CPP (Fig. 4, *P* < 0.05), total porosity under OPP was 7.5% and 10.5% higher than under PFP and CPP, respectively. In the 10–20 cm soil layer, total porosity under OPP was 4.0% and 11.5% higher than under PFP and CPP, respectively. In the 20–40 cm soil layer, there were no significant differences in total porosity across the three conditions. OPP was associated with a high number of continual and stable pores formed by the decomposition of soil animals and microbial activity. In the 0–40 cm soil layer, the proportion of macropores was higher and that of small pores was lower under OPP than under PFP or CPP. The distribution of pore size and structural characteristics were variable, with a high proportion of microporosity and mesoporosity under CPP and PFP treatments.

## Discussion

GMD and MWD are important indicators reflecting the distribution and stability of soil aggregates, where higher GMD and MWD values indicate higher average particle size agglomeration and better soil structure and stability^26,27^. The results indicated that the use of additional organic fertilisers in OPP was associated with an increase in biological waste particles such as plant debris, humus, earthworms, enzyme activity and SOM in greenhouse soils. Moreover, the application of organic materials caused the decomposition of organic matter and plant residues. SOM derived from organic fertilisers is the main cementation agent for soil aggregates and therefore has an important influence on the quantity and size distribution of soil aggregates^28–30^. The humus formed during conversion is an important cementing material for aggregate formation and is beneficial for the formation of large particles, ultimately supporting the content and stability of soil macroaggregates^31–33^. Therefore, it can be concluded that OPP is associated with significant increases in soil aggregate agglomeration and erosion resistance.

The total porosity under OPP was significantly higher than that under PFP or CPP in the 0–10 cm and 10–20 cm soil layers (no difference was found for the 20–40 cm layer) and porosity in each condition was ranked as OPP > PFP > CPP. Under CPP, the main pore types were simple packing voids and surface cracks, with few vughs or channels formed following the decomposition of fresh residues. Soil at 20–40 cm depth was compact with low porosity. Simple pores and surface cracks are primarily found in soils with poor structure and low fertility. In contrast, the PFP soil structure showed simple pores and composite pores, with a small number of holes and pores due to the activity of soil animals and microorganisms. Under OPP, pores were dominated by compound packing voids, with well-developed and intertwined inter- and intra-aggregate pores. A number of pores and vesicles were formed by the decomposition of organic residues and animal activity. Use of organic fertilisers was thus associated with increased porosity in agglomerates, increased recombination in the degree of pores, and improvements in overall soil structure. In this study, OPP had more positive effects on soil quality and structure than PFP. In this long-term fertilisation experiment, with the same amount of nutrient input, more organic fertiliser was applied in OPP than in PFP, therefore, the increase in organic fertiliser likely led to the higher organic matter content in the soil^34,35^. Long-term OPP increased the number of macroaggregates and soil stability, which showed more positive impacts on soil quality and structure than PFP^36,37^. Our results were supported by the results of Xing *et al*.^38^, who found that organic fertiliser application to brown earth increased the macroaggregate content and SOC, and aggregate stability was better than in soil treated with organic and inorganic fertilisers.

Total porosity was higher in the soil surface than in the underlying soil. The growth of crop roots requires sufficient water, nutrients, and space. Large amounts of water and nutrients are stored in large soil pores, while inter- and intra-aggregate pores are particularly important for plant growth. Mesoporosity has some limitations, and microporosity greatly limits the growth of soil roots. The shape and quantity of pores reflect changes in soil structure and soil water movement^39^. Long-term OPP increased the SOM content and improved soil pore distribution, which were verified by studies of Emerson *et al*.^40^. An increase in SOM content results in higher total porosity and lowers soil bulk density^41^. Therefore, OPP is recommended to promote the development of pores, increase soil porosity, optimise the distribution ratio of pores of different sizes, and improve soil structure and crop growth.

**Figure 5:**
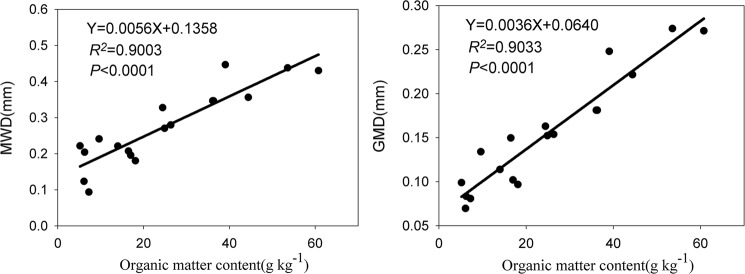
Correlation between soil organic matter content and mean weight diameter (MWD) and geometric mean diameter (GMD).

There were significant positive correlations between SOM content and GMD as well as MWD (Fig. 5; GMD, *R*^2^ = 0.9033, *P* < 0.0001; MWD, *R*^2^ = 0.9003, *P* < 0.0001), which indicated that as SOM increased, the GMD and MWD values of water-stable aggregates also increased, improving the stability of water-stable aggregates and soil structure. These results were supported by the studies of Guber *et al*.^42^ and Wang *et al*.^27^, who found that most of the organic fertilisation practices studied had large MWD values and high amounts of macroaggregates. Therefore, OPP and PFP were associated with significant increases in SOM and effective nutrient content. Accordingly, these practices are potentially effective for improving soil physiochemical properties, fertility, and structural stability in greenhouses of the North China Plain.

## Conclusions

Long-term OPP significantly decreased soil bulk density (1.17 g cm^−3^) while increasing SOM content and total soil porosity (54.8%); the soil surface layer microstructure changed from a moderately separated angular blocky microstructure and mildly separated blocky microstructure to a highly developed crumb microstructure and moderately separated crumb microstructure, with a high content of well-developed aggregates. OPP was also associated with the best GMD and MWD values, which represent a loosening of the soil and greater numbers and stability of large water-stable aggregates. Moreover, OPP promoted the development of aggregate structure and ultimately improved soil aggregate content, as well as soil microstructural characteristics. The pore walls were relatively smooth and individual pores were larger, resulting in a significant increase in porosity of 0.01 mm. In conclusion, compared to CPP and PFP, OPP was associated with significant improvements in soil structure, organic matter, and porosity as well as air permeability, which are favourable for crop growth.

## Methods

### Site description and experimental design

The long-term research site was located at the Quzhou Agriculture and Ecology Experimental Station of China Agricultural University in Quzhou County, Hebei Province, China (36°52′N, 115°01′E). The site had a warm, temperate, semi-humid continental monsoon climate. The annual mean temperature was 13.1 °C, with an accumulated temperature above 10 °C of 4472 °C, with a frost-free period of 210 days and annual average precipitation of 604 mm. A long-term fertilisation experiment was conducted in a greenhouse vegetable field over a 10-year period from 2003 to 2012. In the 0–20 cm soil layer, the basic soil properties were as follows: total N content, 1.24 g kg^−1^; total P content, 1.61 g kg^−1^; available K content, 364.28 mg kg^−1^; and organic matter content, 16.94 g kg^−1^. Basic properties in the 20–40 cm soil layer were as follows: total N content, 0.73 g kg^−1^; total P content, 0.97 g kg^−1^; available K content, 131.18 mg kg^−1^; and organic matter content, 6.89 g kg^−1^.

The planting experiment examined three planting patterns: organic planting patterns (OPP), pollution-free planting patterns (PFP), and conventional planting patterns (CPP). The solar greenhouse for the planting experiment was arched, and each greenhouse was 52 m long and 7 m wide covering an area of approximately 0.04 hm^2^. Each condition was constructed in triplicate. The planting system was a tomato-cucumber rotation system. Texturally, the soil was a clay loam (USDA-NRCS) in which sand, silt, and clay accounted for 22%, 59%, and 19% of the soil, respectively. The soil was classified as Salinized fluvo-aquic soil as per Chinese Soil Taxonomy. Fertilisation time depended on the crop growth stage, weather conditions, and methods of application available, and nutrient application rates corresponded with local agricultural practices. Organic fertilisers applied for OPP and PFP were organically certified, and physical and biological measures were used to control diseases and insect pests during production. Mineral fertilisers were applied for CPP and diseases and pests were controlled using plant protection chemicals. Other cultivation management methods were consistent across the three conditions. The total N, total P, and available K contents of organic fertilisers were measured before fertilisation. Mineral N, P, and K fertilisers included CO(NH_2_)_2_, Ca(H_2_PO_4_)_2_·H_2_O, and K_2_SO_4_. All fertiliser applications were performed based on the total N content of organic planting. Mineral and organic fertilisers were spread evenly onto the soil surface by hand and incorporated into the topsoil by tillage before sowing. Supplemental mineral fertilisers were applied in the same fashion. Detailed planting conditions and the amounts of fertiliser used for different treatments are shown in Table 3.

**Table 3:** Planting patterns and the amounts of fertiliser used for different treatments (kg hm^−2^).

Planting pattern	Fertilisation method	Spring			Autumn			The whole year		
		N	P_2_O_5_	K_2_O	N	P_2_O_5_	K_2_O	N	P_2_O_5_	K_2_O
Conventional planting	100% chemical fertilisers	426.2	375.0	605.0	293.8	75.0	285.0	720	490	890
Pollution-free planting	70% organic + 30% chemical fertilisers	337.2	305.5	344.3	382.8	154.5	565.7	720	460	910
Organic planting	100% organic fertilisers	248.1	246.0	113.7	471.9	234.0	843.0	720	480	960

### Sampling and measurement methods

Disturbed and undisturbed soil samples were taken at depths of 0–10, 10–20, and 20–40 cm from each plot with a 10-cm diameter soil corer in early September 2013 after the cucumber harvest. Six cores were sampled in each plot (three disturbed and three undisturbed cores were taken adjacent to one another). We minimised soil disturbance during collection and transport to avoid disrupting the soil aggregate structure. Hardened blocks of soil samples were cut into 6 cm × 2 cm × 30 µm thin sections for microscopic observation.

Soil samples for bulk density determination were taken with a 100 cm^3^ metal ring and oven dried at 105 °C. Soil density was measured using the pyknometer method. Total porosity was calculated from the soil bulk density and particle density. Organic carbon content was measured by the rapid dichromate oxidation method^43^. Soil organic matter (SOM) content was calculated by multiplying the soil organic carbon content by a factor of 1.724^44^. Particle size was measured by the hydrometer method^45^ with solidification of soil micromorphological samples as described by Murphy^46^. The wet sieving method was used to investigate soil aggregate stability and the size distribution of water-stable aggregates^47^. The aggregate content fraction >0.25 mm (*R*_0.25_), mean weight diameter (MWD), and geometric mean diameter (GMD) were calculated using Equations 1, 2 and 3, respectively^48,49^.

1 $${R}_{0.25}=\frac{M(r > 0.25)}{{M}_{T}}=1-\frac{M(r\le 0.25)}{{M}_{T}}$$

2 $${\rm{MWD}}=\frac{{\sum }_{i=1}^{n}(\overline{{x}_{i}}{w}_{i})}{{\sum }_{i=1}^{n}{w}_{i}}$$

3 $${\rm{GMD}}=\exp (\frac{{\sum }_{i=1}^{n}{w}_{i}\,\mathrm{ln}\,\overline{{x}_{i}}}{{\sum }_{i=1}^{n}{w}_{i}})$$

where n denotes the number of aggregate size fractions, $\overline{{x}_{i}}$ is the mean diameter of aggregates retained in the *i*th sieve, *W*_*i*_ is the aggregate weight retained in the *i*th sieve, *M*(*r* ≤ *x*_*i*_) is the weight of aggregates with a fraction diameter less than or equal to *x*_*i*_, and *M*_T_ is the gross weight of aggregates. According to multilevel aggregate formation theory, macroaggregates with diameters of 0.25–10 mm represent a granular structure (*R*_0.25_), which is the best structural aggregate in soil^50^.

### Soil thin section micromorphological observation and analysis

Micromorphological study of soil thin sections was performed using a polarising optical microscope (LV100 POL, Nikon, Tokyo, Japan). The micromorphological features examined in each thin section were based on Stoops^26^. Plain polarised light (PPL) and cross-polarised light (CPL) TIF images of each section were captured by a digital camera (Nikon DS-Fi1). Images were 50 × magnification with a resolution of 4.5 µm pixel^−1^. The dimensions of each image were 2560 × 1920 pixels^2^, equivalent to approximately 1.10 × 0.76 mm^2^. The “S” method was adopted for photographing, and 20 points in a soil thin section were selected to provide a statistically representative area^51^.

All images were analysed and processed using Matlab and Photoshop software on a personal computer. Pore space and shape data were extracted and measured in accordance with Mooney *et al*.^52^. CPL images were taken from 0° and 45° directions in the same field to avoid the overestimation of pore area caused by transparent minerals. Then, the pore area was obtained by subtracting the binary composite CPL image from the binary PPL image. In the process of soil thin section production and image capture, the effects of noise were unavoidable; therefore, we also enhanced and denoised the pore space in order to accurately obtain information on pores. Pore types were classified as microporosity (<20 µm), mesoporosity (20–100 µm), and macroporosity (>100 µm). Macroporosity and mesoporosity were quantified directly using Matlab and Photoshop software from the images of soil thin sections, whereas microporosity was calculated as the difference between total porosity and macroporosity plus mesoporosity.

### Statistical analyses

Data analysis and figure generation were performed using Microsoft Excel 2016 and SigmaPlot 10.0. A one-way analysis of variance was performed to identify differences in soil bulk density, SOM, MWD, GMD, and porosit.y among the different planting patterns. Duncan’s multiple range test was adopted to test differences between the different planting conditions using SPSS 22.0. A *P-*value < 0.05 was statistically significant. Some micromorphological features were described qualitatively and compared by careful observation of soil thin sections.
